# Knee Pain as the Reason for Encounter in General Practice

**DOI:** 10.5402/2013/930825

**Published:** 2013-12-26

**Authors:** Thomas Frese, Linda Peyton, Jarmila Mahlmeister, Hagen Sandholzer

**Affiliations:** Department of Primary Care, Leipzig Medical School, Philipp-Rosenthal-Straße 55, 04103 Leipzig, Germany

## Abstract

*Objective*. Currently, an overview of the management of knee pain in general practitioner's offices is not available. The main concern of this study was to evaluate the consultation prevalence of knee pain, accompanying symptoms, the frequency of diagnostic and therapeutic procedures, and results of encounters of patients suffering from knee pain. *Methods*. For the SESAM 2 study cross-sectional data was collected from randomly selected patients during one year and compared with publicly available data from the Dutch Transition Project. *Results*. Overall, 127 out of 8,877 (1.4%) patients of the SESAM 2 study and 6,754 out of 149,238 (4.5%) patients of the Dutch Transition Project consulted for knee pain. Drug prescription, follow-up consultation, giving doctor's advice, and referral to a specialist or physiotherapist were the most frequent procedures. Osteoarthritis of the knee and other musculoskeletal diseases were the most frequent results of encounter. Overweight, age, gender, and other musculoskeletal diseases were found to be significantly associated with knee pain. *Conclusion*. Knee pain in general practice settings is mainly associated with chronic problems. Dangerous outcomes (as suspected fracture or thrombosis) are rare. Further research is needed in order to reduce the influence knee pain has on daily living.

## 1. Introduction

Knee pain is a common complaint and is therefore a typical reason for consulting the general practitioner. Knee pain occurs increasingly with advanced age [1] and among other issues therefore plays an important role in an aging population, like ours [2]. Already, approximately 25% of the people aged over 55 years suffer from constant knee pain [3] which can have a negative effect on the quality of life [4]. The diagnosis of knee pain requires a comprehensive evaluation including medical history taking, specific physical examination using manual diagnostic tests, and medical imaging. Clinical guidelines for the systematic examination of the knee [5], history taking [6], and treatment options are available for physicians. There are multiple causes of knee pain and, conclusively, manifold treatment options [7].

Currently, an overview of the management of knee pain in general practitioner's offices is not available. The main concern of this investigation was to evaluate the consultation prevalence of knee pain, the frequency of diagnostic and therapeutic procedures, accompanying symptoms, and results of encounters of patients suffering from knee pain. Therefore, data from the German SESAM 2 study and the Dutch Transition Project [8] were analysed.

## 2. Methods

All general practitioners in Saxony were contacted by the Saxon Society of General Medicine (SGAM). They received no incentive for their participation. The study was designed to document reasons for encounter, diagnostic and therapeutic procedures and the result of the encounter (chosen diagnosis).

Of the 2,510 general practitioners that were contacted, 270 agreed to participate and 209 cooperated for the entire study period of one year. Cross-sectional information was collected from October 1, 1999 until September 30, 2000. Case recording was carried out on one day a week (Monday to Friday; either morning or afternoon consultation hours), chosen at random. Data was collected for one of ten patients previously known to the practitioner. Multiple recording of the same patient was avoided. House calls were not considered. A total of 8,877 patients were included. A standardized data collection form was used [9]. It was developed by general practitioners (Leipzig Medical School and Saxon Society of General Medicine). The form was tested and evaluated during a pilot trial (SESAM 1). Each patient's reasons for encountering their general practitioner, symptoms, diagnostic procedures, and results of encounter “diagnoses” as well as general morbidity and therapeutic procedures were documented. As far as possible, data was recorded verbatim (according to the study instructions), either as told by the patient (e.g., reasons for encounter) or in the physician's words (e.g., chronic diagnoses). Due to the random selection, the information was documented in a reasonably short time. Only completely filled-in forms were considered.

As described elsewhere, the SESAM 2 study provides independent and unbiased cross-sectional data from a daily primary care setting [10, 11]. Since the total morbidity was estimated there is no selection bias and the data can be assumed to be representative. The International Classification of Primary Care (ICPC) version of 1987 was used to code the reasons for the encounter [12]. The reason of encounter was encoded as L15 (knee symptom/complaint). The German SESAM 2 data was analyzed and compared to the unpublished but publicly available data from the Dutch Transition Project (described by Lamberts and Okkes [13]; total estimation of patients from about 20 general practitioners; from 1985 till 2003). The data is available at http://www.transitieproject.nl/ and can be analysed using the software that is contained within the database. The performance of the SESAM 2 study was in accordance which the guidelines of the Institutional Review Board/Ethics Committee. As stated by the Ethics Committee no special approval was demanded.

Statistical analysis of the SESAM 2 data was performed using Statistical Packages for Social Sciences (SPSS 15.0; SPSS Inc., Chicago, USA). As indicated, data was compared using Fisher's exact test and *χ* ^2^ test. Differences were stated as statistically significant for *P* < 0.05.

## 3. Results

Within the SESAM 2 study 209 general practitioners participated and 8,877 patients were reported by them. Of those 5,050 (56.9%) were females, 3,824 (43.1%) were males, and 3 patients were not specified. During the study period 127 patients consulted their general practitioner because of knee pain. This means a consultation prevalence (cp) of 1.4%. Of the 127 patients 64 (50.4%) were females.

The Dutch Transition Project contains data from 149,238 as active listed patients. Of those 84,285 (56.5%) were females. A total of 6,754 (4.5%) patients, thereby 3,630 (53.8%) females, consulted their general practitioner because of knee pain. There were 5,430 patients consulting for newly occurred and 2,234 for previously known knee pain during the study period. The distribution of the patients consulting for knee pain among different age groups is given in Table 1. There were 5,675 consultations for newly occurred and 2,816 for previously known knee pain during the study period.

The Dutch Transition Project revealed that at the age range of 15 to 44 men suffer more frequently from new knee pain (*P* < 0.01 for each age group). This was also the case for previously known knee pain at the age range from 25 to 44 years. In contrast, at the ages of 65 to 74 years, women were statistically significantly more affected by new and previously known knee pain (*P* < 0.01 for each) and suffered more frequently from knee pain at an age over 75 (*P* < 0.01) than men.

The diagnostic and therapeutic procedures that were performed in patients that consulted for knee pain are summarized in Table 2.

We compared the frequencies of results of encounter of patients consulting for knee pain to those of those without knee pain. As indicated in Table 3, the most common “diagnosis” associated with knee pain was osteoarthritis. Knee pain patients were affected five times more often than the general patient population. In the group of knee pain patients 12.6% additionally were documented to suffer from overweight compared to the general patient population with only 6.9%.

Table 4 shows the most common results of encounter among patients with knee pain during the SESAM 2 study in comparison to the results from the Dutch Transition Project. The most frequently diagnosed cause of knee pain found during the SESAM 2 study was osteoarthritis of the knee followed by other injuries of the musculoskeletal system. Comparative in the Dutch Transition Project the most frequent diagnosis was “knee symptoms” in general (43.9% for newly occurred knee pain versus 32.8% for previously known knee pain). During the SESAM 2 study (*n* = 127) and within the group of consultations for newly occurred knee pain from the Dutch Transition Project (*n* = 5,430), six patients had a suspected thrombosis and two a suspected fracture of the tibia or fibula.

## 4. Discussion

Our study confirmed that knee pain is a regularly occurring reason for encounter in general practice. Women at an older age suffered from knee pain significantly more often than men. The most common reason for knee pain at advanced age was osteoarthritis of the knee.

Since SESAM 2 there has not been any new relevant published data from cross-sectional studies regarding knee pain in general practice. The SESAM 2 study showed that with a consultation prevalence of 1.4% patients with knee pain represent a respectable group in general practice; however, this lays markedly below the overall prevalence in the general population: it is assumed that more than 25% of our society suffers from constant knee pain. The group of patients with knee pain who consult their general practitioner is quantified with 25% [14]. Among others this disparity is presumably attributed to the fact that patients with knee pain often do not consult their general practitioner but a specialist [15]. In the case that 25% of the population suffer from knee pain and a quarter of those consult their general practitioner a consultation prevalence of 5% is received. Therefore, the consultation prevalence found in the Dutch Transition Project (4.5%) is in line with the upper assumptions regarding the prevalence of knee pain in the general population. Furthermore, it has to be mentioned that in The Netherlands the general practitioner is always the first point of contact “gate keeper” for patients. This might explain the different consultation prevalence found in the SESAM 2 study and the Dutch Transition Project.

The recent data suggest that men younger than 65 years do suffer more often from knee pain than age matched women. This might be a result of sports related injuries and chronic articular changes [16]. Women show an increasing prevalence with advancing age, which correlates with the expected development of chronic diseases of the joint [17]. This leads to the reported higher consultation prevalence for knee pain in women older than 74 years compared to age matched men.

Knee pain is occasionally accompanied by other symptoms, which particularly affect the musculoskeletal system (especially, hip, legs, feet, back, shoulders, and hands) in the form of pain, stiffness, and swelling. Furthermore, unspecific symptoms such as fever, fatigue, and weakness rarely appear.

In a general practice setting the anamnesis [18, 19] and physical examination can be assumed to play an outstanding role in diagnosing knee pain. This is underlined by the reported findings that illustrate a high frequency of physical examination of knee pain patients in general practice (Table 2). However, the results of both studies stand in contrast with already existing recommendations, saying that taking history (including first impression) is the most important diagnostic tool. Special knee tests and diagnostic imaging are additional aids [20].

In a general practitioner setting knee pain is most commonly caused by disorders of the knee and the lumbar spine. Seldom occurring reasons of knee pain, such as psychological and neurological diseases, play a role (Table 4). Dangerous courses are rare. The age of the patient can be a factor when attempting to rule out certain diagnoses [6]. With children, adolescents, and young adults, for example, the most common reasons are leisure or work accidents, sports injuries, and seldom rheumatic diseases. With progressing age chronic processes of the knee joint in the form of arthritic changes up to arthrosis are the leading cause of knee pain [21]. In rare cases the reason for knee pain has to be urgently investigated and treated. If the pain occurs acutely fractures and thromboses have to be ruled out and in case of fever the general practitioner has to consider a severe inflammation. Based on the age distribution (>60% of the patients were older than 45) gonarthrosis and other musculoskeletal disorders were most common. Correlating with the weight development of our society's population the share of obese patients was 12.6% [22]. Overweight is an important risk factor for joint diseases and was found among others to be significantly associated with the occurrence of knee pain [23]. Further frequently accompanying diagnoses are osteoarthritis of the knee and other joints. Within the Dutch Transition Project knee symptoms and osteoarthritis were the most frequent results of encounter. The differences between both studies might be explainable by the varying coding systems and coding customs in Germany and The Netherlands.

The most common therapeutic procedures were the prescription of drugs followed by physical therapy and doctor's advice. Most of the therapeutic procedures were applied much less frequently in the Dutch Transition Project than they were assessed during the SESAM 2 study. This can be caused by the fact that the documented procedures in the SESAM 2 study must not necessarily be related to knee pain (e.g., laboratory investigations due to the control of diabetes mellitus as a coencounter). Another possible cause might be found in the study design. During the SESAM 2 study only individual cases were ascertained and the patient as well as doctor was aware of the special situation. This might have led to behavioural changes of the doctor towards a social desirable behaviour. Within the Dutch Transition Project all patients were ascertained regardless of the individual. Encoding “doctor's advice” as a precise procedure is not possible using the ICD-10 as it was used during the SESAM 2 study but explicitly with the ICPC-2 used during the Dutch Transition Project. This circumstance might have led to neglecting documentation of this procedure during the SESAM 2 study and be jointly responsible for the remarkably big difference in frequency comparing the two studies.

Porcheret et al. [24] developed an evidence-based case model for the treatment of knee pain in a primary care setting. It is a four-step instructional plan that on the first two levels suggests putting the patient in charge of himself and letting him or her undertake responsibility. It is called the self-care level and includes primarily weight reduction, exercise, thermotherapy, physiotherapy, and the intake of over-the-counter drugs, such as acetaminophen. Besides the conventional therapeutic procedures the field of alternative and complementary medicine provides a wide range of treatments for knee pain [25, 26]. These however were not specifically considered during the SESAM 2 study or in other general practice studies dealing with knee pain. Basis of “self-care” is a comprehensive consultation of and information on the disease, its progress, and treatment options. This information should be delivered by the general practitioner both orally and in writing. This way the patient is supposed to be put in an autonomous situation in which he or she can decide for him- or herself the course of treatment that he or she finds appropriate.

In summary, we conclude from our findings that knee pain in general practice settings is mainly associated with chronic problems. Dangerous outcomes (as suspected fracture or thrombosis) are rare. Further research is needed in order to reduce the influence knee pain has on daily living.

## Figures and Tables

**Table 1:** Patient distribution (pd) and consultation prevalence (cp) of knee pain in different age groups in the German SESAM 2 study and the Dutch Transition Project regarding new and previously known knee pain.

Age (years)	SESAM 2 *n* = 127			DTP (new) *n* = 5,430			DTP (known) *n* = 2,234		
	*n*	pd (%)	cp (%)	*n*	pd (%)	cp (%)	*n*	pd (%)	cp (%)
0–4	0	0.0	0.0	28	0.5	0.2	2	0.1	<0.1
5–14	1	0.8	0.3	460	8.5	3.1	86	3.8	0.5
15–24	14	11.0	1.6	823	15.2	5.0	283	12.7	1.7
25–44	23	18.1	1.3	1698	31.3	3.7	570	25.5	1.2
45–64	42	33.1	1.4	1404	25.9	4.2	616	27.6	1.8
65–74	26	20.5	1.5	645	11.9	4.0	359	16.1	2.2
75+	21	16.5	1.8	372	6.9	2.5	318	14.2	2.1

Total	127	100	1.4	5430	100	3.6	2234	100	1.4

new: newly occurred knee pain, known: previously known knee pain.

pd: patient distribution, refers to the group of knee pain patients.

cp: consultation prevalence, refers to all patients of the same age group within the study population.

**Table 2:** Procedures performed during the consultation of patients for knee pain (%) in the German SESAM 2 study and diagnostic and therapeutic procedures in the Dutch Transition Project regarding new or previously known knee pain.

Procedure	SESAM 2	DTP (new)	DTP (known)
	*n* = 127	*n* = 5,675	*n* = 2,816
Examination	92.1	94.2	82.6
Follow-up consultation	74.8	n.a.	n.a.
Drug prescription/injection	59.1	16.3	21.5
Referral	52.2	5.8	17.8
Physical therapy	43.3	11.5	16.6
Incapacity for work	24.4	n.a.	n.a.
Doctor's advice	14.2	50.3	33.6
Diagnostic imaging	12.8	10.5	13.2
Laboratory	11.0	1.3	1.6
Dressing/compression/packing	7.9	2.9	1.2
Exclusively taking history	4.7	n.a.	n.a.
Incision/drainage/aspiration	3.1	0.3	0.9
Hospitalization/emergency referral	2.4	0.3	0.2
Local injection/infiltration	1.6	0.9	1.2
Other diagnostic procedures	0.8	0.3	1.1
Other therapeutic procedures	1.6	0.3	0.7

n.a.: not assessed.

**Table 3:** Significantly associated results of encounter (%) found among knee pain patients (*n* = 127 with knee pain) compared to other patients (*n* = 8,750) within the German SESAM 2 study.

Result of encounter	Patients with knee pain	Other patients	*P* (Fisher)
Osteoarthritis of the knee	44.9	8.6	<0.001
Other musculoskeletal diseases	33.9	1.1	<0.001
Overweight	12.6	6.9	0.021
Rheumatic polyarthritis	9.4	2.4	<0.001
Acute injuries	8.7	3.3	0.004
Atherosclerosis	2.4	0.6	0.042
Malformations/deformities	0.8	0.0	0.042

**Table 4:** Total morbidity (%) found among knee pain patients (based on the International Classification of Primary Care; Chapters A, L, N, P, and T) of the German SESAM 2 study (*n* = 127 patients with knee pain) and the Dutch Transition project (DTP) for newly occurred (*n* = 5,430) and previously known (*n* = 2,234) knee pain.

Result of encounter (ICPC-2 code)	SESAM 2	DTP (new)	DTP (known)
Osteoarthrosis of knee (L90) or osteoarthritis (L89)	47.2	9.4	27.9
Other musculoskeletal diseases (L99)	32.3	6.1	5.5
Obesity (T82)	12.6	<0.1	<0.1
Other osteoarthrosis (L91)	7.9	0.0	0.0
Varicose veins of leg (K95, excel. S97)	7.1	0.4	0.3
Other injury of the musculoskeletal system (L81)	3.9	6.5	3.7
Osteoarthritis of spine (L84)	3.9	<0.1	0.1
Inflammation of joint, tendonitis (L87)	3.9	0.2	<0.1
Effect of prosthetic device (A89)	3.1	0.0	0.0
Sprains/strains of knee (L78)	3.1	8.7	6.7
Rheumatic arthritis (L88)	3.1	0.1	0.9
Lumbar disc lesion/radiation (L86)	2.4	0.2	0.6
Osteoporosis (L95)	1.6	<0.1	<0.1
Hypochondriacal disorder (P75)	1.6	<0.1	0.1
Peripheral neuritis/neuropathy (N94)	0.8	0.0	0.0
Knee symptoms/complaints (L15)	—	43.9	32.8
